# A Time to Die: Mexican Life Debates and the Paradox of Choice in Palliative Care

**DOI:** 10.1007/s11013-025-09937-0

**Published:** 2025-10-05

**Authors:** Elyse Ona Singer, Norma Alicia Ordóñez Vázquez

**Affiliations:** Southern Methodist University, Dallas, USA

**Keywords:** Palliative care, Dignified death, Life debates, Choice, Mexico

## Abstract

Enduring contests between church, state, and individual control over the body in Mexico have recently manifested in a contentious “life debate” over the definition and ethics of “dignified death” (Roberts in God’s laboratory: assisted reproduction in the Andes. University of California Press, 2012). In 2008, Mexico City passed the Advance Directive Law, authorizing the right to refuse or withdraw life-sustaining treatment and affording palliative care services to the terminally ill through public hospitals. While these measures have ostensibly ameliorated end-of-life suffering, a growing movement of activists argues that the law falls short. They advocate for the legalization of assisted dying as central to “dignified death” despite resistance from the Catholic hierarchy and its political allies. Drawing on one year of research in Mexico’s National Cancer Institute, we analyze the place of public palliative care clinicians in this fraught debate, parsing their model of care, which is predicated on surrendering to the body’s preordained rhythms of life and death. We argue that this orientation gives rise to a paradox at the heart of public palliative care, which simultaneously expands end-of-life choices while making certain choices inconceivable. Our argument has implications for anthropological theories of human agency and choice, provoking complex questions about who is authorized to draw boundaries around life and death.

## Introduction

A family stands around the bed of Araceli,^1^ a young woman who is dying of gynecological cancer. Inside a bedroom in her home, the light is dim. One of Araceli’s children plays the guitar while the other sings to her. Her husband kisses her cheek as she smiles back at him. She looks frail as she lies in bed, but also peaceful, her pain under control. She has opted out of all curative measures and is receiving only palliative care. A sign on the wall behind her head reads “Please don’t come here to cry, but instead to accompany Araceli.” Everyone present, including Araceli, knows and seems to have accepted that she is dying. Araceli will spend her final days being ushered out of the world calmly, surrounded by her loving family, without resistance, denial, or regret.

This is the scene that Dr. Molina, a palliative care doctor at Mexico’s National Cancer Institute (INCan), rendered when asked to recall a case of one of her patients whose death she considered “dignified.” As an out-patient palliative doctor in the public sector, Dr. Molina had not been present at this scene, or when Araceli eventually passed away. One of Araceli’s daughters had videotaped her mother’s final days and sent it to Dr. Molina as reassurance that her mother had been comfortable, and to thank her for caring so attentively for her in the preceding months. Of course, not all palliative care patients at the INCan die this way. In fact, Dr. Molina had been so moved by the serenity of Araceli’s death scene that she had saved a copy of the video on her work computer as a model of the kind of death to which she and her colleagues on the palliative care ward could aspire to occasion for all their patients.

As a pair of medical anthropologists, we first arrived on the INCan’s palliative care ward in the summer of 2023 as part of a larger ongoing anthropological study of “dignified death”^2^ in the Mexican capital.^3^ We wanted to understand the implications of Mexico City’s 2008 Advance Directive Law (ADL), which afforded the right to refuse or withdraw life-sustaining measures. As part of the law, all capital residents may now register their end-of-life wishes by filing advance directives at a hospital or public notary, and comprehensive out-patient palliative care services have been made available to the terminally ill for the first time through several public health institutions (Álvarez Del Río, 2009). The first law of its kind in the country, the Mexico City ADL was designed to guarantee “death with dignity,” preventing the widespread practice of what is called “therapeutic obstinacy,” the relentless use of biomedical interventions at the end of life that offer little significant benefit to patients. Over 20,000 capital residents have since filed advance directives, and similar laws have been passed in thirteen other Mexican states (Gómez, 2023).

While the Mexico City ADL has ostensibly expanded patient choice and diminished suffering at the end of life, a local movement of activists contends that the law falls short. These activists have made the right to assisted dying^4^ central to their advocacy work, arguing that individuals who want immediate relief from debilitating or incurable conditions should be allowed to end their suffering as a central component of “dignified death” (Espinosa, 2016; Mansilla-Olivares et al., 2018). Even as legislation on euthanasia has become more permissive across Europe, North America, and Australasia, the practice remains criminalized in Latin America, with the exceptions of Colombia and Ecuador. While euthanasia is illegal in Mexico, individuals who can afford to hire private doctors can access the procedure in the private sector for a fee; but those with fewer resources are generally unable to access this privileged form of dying. In their efforts to legalize euthanasia, activists have encountered tremendous resistance from the Catholic hierarchy, which bears heavily on political and popular life in Mexico despite the secular foundations of the Mexican state. While the refusal of futile medical interventions protected in the ADL is considered congruent with Catholic doctrine, Catholic officials oppose assisted dying, framing it as an assault on human dignity and a violation of God’s authority over life (CCC 2324).^5^

Anthropologists have long considered cultural and political contestation at the thresholds of life and death (Das & Han, 2016; Morgan, 2009; Timmermans, 1999). These liminal periods can be exceptionally charged as “[the] margins of life [are] a site for examining the making and unmaking of persons and relationships, social and corporal bodies, and life itself” (Morgan and Kaufman, 2005, p. 318). Elizabeth Roberts’ (2012) concept of “life debates” helps to elucidate the impassioned legal, ethical, and political disputes unfolding over how to regulate the boundaries of life and death in contemporary Mexico, reflecting enduring contests between church, state, and individual control over the body. Our research revealed that palliative care clinicians are curiously situated in the ongoing national “life debate” over “dignified death,” harboring profound reservations about assisted dying (Roberts, 2012). Although we had anticipated that their ready exposure to extreme human suffering would make them easy allies with “dignified death” activists, as one doctor remarked, “as palliative care professionals, we are in favor of life.” Such comments caught our attention on a medical ward dedicated to accomplishing “dignified death,” even if a degree of moral uncertainty about assisted dying is palpable in the wider context of Mexico.^6^ We wondered why, in the space of Mexican palliative care, life was an ultimate good worth preserving,^7^ and how such an orientation figured in efforts to accomplish a kind of death that can be considered “dignified.”

We are interested here in how palliative care providers in one public health institution in the Mexican capital work to orient terminally ill patients to mortality in particular ways to accomplish their vision of “dignified death,” and what happens when this process of attunement falters. Over the course of a year of ethnographic research on the ward, we came to understand that two seemingly conflicting imperatives defined the model of care at the INCan.  Providers sought to foster in dying patients both acceptance of death and a will to live. They wanted to induce awareness of death, while creating the conditions in which the desire to hasten death did not arise. We argue that this orientation gives rise to a paradox at the heart of Mexican palliative care, which both expands end-of-life choices, while also positioning itself against the logical implications of choice itself, making certain choices inconceivable. Our argument has implications for anthropological theories of human agency and choice, provoking complex questions about who is authorized to draw boundaries around life and death.

To build our argument, we begin by reprising anthropological literatures on care for the dying and then describe the study from which these data are drawn as well as the ethics of this sensitive research. We proceed to delve more deeply into philosophies of care on the ward. Next, we relay two stories that represent either pole of the spectrum of problematic death according to the clinicians: that of a patient we call Karo, who struggled to accept her mortality, evincing what the clinicians labelled “death denial,” and that of a patient we call Alejandro, who was eager to die before his body was ready, and whom several clinicians suspected had taken his own life. Analyzing the cases of these two patients, whose orientation to death challenged the clinicians’ approach and caused them tremendous moral distress, allows us to parse the limits of agency and choice available in Mexican palliative care.

## “Dignified Death,” Palliative Care, and the Limits of Choice

Although humans everywhere must confront the reality of death, anthropologists, and other social scientists observe that how it unfolds, as well as understandings about what constitutes a “good” or “bad” death, are culturally situated (Arkin, 2020; Buchbinder, 2018; 2020; Clive and Van der Geest, 2004; Koksvik, 2020; Hannig, 2022; Nishimura et al., 2022; Norwood, 2020; Stonington, 2015). At the same time, most people agree that dying peacefully and without pain while surrounded by family after a long and vibrant life is better than dying alone, violently, or prematurely (Clive and Van der Geest, 2004). Moreover, research suggests that most people want biological death to precede “social death,” so that their bodies give out before their personhood, social roles, and relationships unravel (Biehl, 2005; Buch, 2013; McNamara, 2004; Norwood, 2020).^8^ While trying to pin down a definition of “dignified death” is challenging, one thing is certain: although death comes to everyone, it comes in vastly uneven ways, representing what critical medical anthropologist Merrill Singer et al. (2025, p. 79) call “the embodiment of inequality.” In Mexico, where wealthy individuals can pay to access criminalized forms of privileged death, this is undoubtedly true.

One factor that can make the difference between dying well or badly is the availability and quality of end-of-life care. Care for the dying poses questions about the degree to which personhood and dignity can be sustained amidst cognitive and physical decline, the erasure of status-conferring social roles, pain and discomfort, as well as losses of autonomy and productivity (Biehl, 2005; Buch, 2018(Rouse, and Rouse, 2008)). The movement of death from home to hospital during the twentieth century has had profound implications for end-of-life care, transferring the duty of care from kin to biomedical professionals. While the medicalization of death has allowed for more control over an otherwise unpredictable process, research suggests a more ominous reality: what medical anthropologist Sharon Kaufman (2005, p. 4) describes as “a prolonged hovering at the threshold between life and death.” In the era of medicalized death, to be sure, many people have begun to question the biomedical imperative to preserve life at all costs when meaning, depth, and quality are compromised (Kaufamn, 2005; Kaufman & Fjord, 2011; McNamara, 2004; Van der Geest, 2004). For physicians in one US hospital, care for the dying entailed letting a patient slip away rather than extending “body time” when “life time” had clearly run out (DelVecchio-Good et al., 2004).

Palliative care “[serves], to some extent, as a symbolic critique of how dying people are managed in highly medicalized settings,” offering an alternative to standard biomedical care (McNamara, 2004, p. 930). Since its emergence in the UK and the US in the 1960s, palliative care has become an integral part of efforts to enact “dignified death” worldwide, although severe access issues persist, particularly in low and middle-income countries (Knaul et al., 2018; Samuels & Dekker, 2023). While definitions vary, palliative care seeks to increase quality of life in people with chronic and terminal illness by mitigating physical, psychological, social, and existential suffering, what the discipline’s founder Cicely Saunders labeled “total pain” (Fajardo-Chica, 2020). Organizational associations such as the International Association for Hospice and Palliative Care (IAHPC) describe a model intended to palliate symptoms of disease and/or treatment in patients and their family members *without* hastening or postponing death. At the same time, research shows that there is not always consensus among palliative care clinicians on how best to care for the dying. Clinicians in one study based in Australia strove for “good enough death,” prioritizing pain reduction while falling short of meeting all their patients’ end-of-life wishes (McNamara, 2004). Moreover, as palliative care globalizes, its meanings, arrangements, and form are inflected with local cultural values and moral concepts (Samuels & Dekker, 2023). The recent legalization of euthanasia in several jurisdictions has also transformed what it means to care for people as they die, complicating the definition and role of palliative care. In some countries, palliative care professionals offer assisted dying as part of the ambit of palliative care, or else refer requesting patients for these services, whereas in other countries, palliative clinicians openly object to assisted dying, defining it as antithetical to their work (Gerson et al., 2020).

While new forms of care at the end of life would seem to protect dying patients from paternalistic medical systems, expanding their options and reinforcing their autonomy, a growing body of work in medical anthropology parses the limits and contradictions of choice in these medical domains. Buchbinder (2020) observes that the legalization of assisted dying in Vermont has afforded a degree of agency and control over death, but only for individuals who possess the economic and cultural capital to gain access to this extraordinarily privileged form of dying. Working in the French context, Arkin (2020) considers how palliative care providers invoke the concept of autonomy to defend their work against medical paternalism even as they guard the limits of which patient choices are considered legitimate. Reflecting critically on his dual role as a palliative care physician and anthropologist, moreover, Stonington (2015) has contemplated how patient desires may contradict biomedical imperatives. He asks what palliative medicine might learn from dying patients who choose to experience, rather than eliminate, pain, as a meaningful and unavoidable part of the dying process. Building on this literature, we consider how local values, religious commitments, and cultural dynamics inflect efforts to enact “dignified death” in Mexican palliative care in ways that both expand and constrict the choices available to dying patients.

### Research and Ethics on the Palliative Care Ward

The data presented here are drawn almost entirely from the palliative care ward of Mexico’s National Cancer Institute (INCan), which serves thousands of patients from across the country every year.^9^ While the hospital primarily serves low-income patients without formal employment or social security, the population is diverse and includes those from a variety of class and regional backgrounds.^10^ Cancer diagnosis often occurs late across Latin America due to lack of investment in cancer prevention and care, fragmented health systems, and educational barriers. For these same reasons, many patients arrive at the INCan with advanced stages of disease (Rodríguez-Mayoral et al., 2019). As the first palliative care ward for cancer patients in the country, the INCan service is known as a national leader, providing out-patient pain management, psychological services, as well as social and existential support. The INCan also provides a residency program in palliative medicine and a diploma in oncological palliative care. Because it is a key training site, clinicians regularly emphasize, both verbally and behaviorally, aspects of care that define their philosophy of healing, making it an ideal research site. While the INCan is considered among the best public palliative care wards in the country, as part of a public institution, resources, and medications, including fentanyl, inevitably run out. Individuals who can afford to pay for palliative care in the private sector will enjoy access to more resources, more luxurious hospital infrastructure, and, ultimately, more end-of-life choices.

To streamline patient care, the INCan palliative care ward is divided into four consultation rooms organized by type of cancer. Each consultation room has an assigned doctor who is the primary medical point person for the patient, as well as a nurse, psychologist and/or psychiatrist, and a rotating group of medical residents undergoing training in palliative care. The palliative service also has a nutritionist, a dentist, and, more recently, a physical therapist. In the INCan model, care is distributed between the palliative team, which works to control physical, psychological, existential, and social symptoms of terminal cancer and cancer treatment; the patient, who is responsible for keeping a written log of their symptoms to help the team adjust treatment; and the patient’s primary family caregiver (a spouse, adult child, parent, sibling, friend, or chosen kin), who bridges hospital care with care at home.

This research was designed to mirror the arrangement of care on the ward. We followed the cases of 48 terminally ill patients receiving palliative care (32 men and 14 women) along with their primary family caregiver, from the time of recruitment until their passing. Case study participation included one in-depth interview with the patient, as well as a separate interview with the family caregiver at the time of recruitment, and a second interview with the family caregiver after the patient passed away, if they were up for speaking (only six family members in the INCan sample agreed to participate the second interview). Ethnographic observations on the ward also formed a central dimension of this research. Embracing the perspective in medical anthropology that the clinic is a space where “the core values and beliefs of a culture come into view,” we embedded ourselves in daily routines on the palliative care ward, observing clinicians across the four consultation rooms about three days a week for one year between July 2023—June 2024 (van der Geest & Finkler, 2004, p. 1995); Long et al., 2008). Finally, to understand biomedical notions of “dignified death,” we interviewed 25 palliative care providers.^11^

Research of any kind with dying people raises serious ethical and emotional concerns related to participants’ ability to consent to research given their deteriorating state and the potentially invasive presence of the researcher during a stressful, sensitive, and painful process (Krawczyk, 2017; Lawton, 2001; Lemos Dekker, 2019).^12^ We were clear during recruitment that study participation was completely voluntary and that refusing to participate would have no bearing on the care individuals received in the hospital. Across this research, we sought to enact a variant of what medical historian Adria L. Imada (2022, p. 27) calls an “ethics of restraint,” whereby the researcher exercises prudence when soliciting patient narratives on emotionally charged and potentially sensitive aspects of human experience as an ethical move to protect human subjects.^13^ Although we spoke directly with patients and their kin, soliciting their narrations of end-of-life experience, in all our conversations we followed participants’ lead toward and away from sensitive topics, *restraining*, in many cases, from asking directly about death and dying when doing so risked jeopardizing a patient or family member’s process of coming to terms with terminal illness. In this way, we strove to mirror the restraint enacted by palliative care clinicians, who treaded carefully with patients, gently inviting them into hard conversations and respectfully pulling back when they sensed the patient was not ready. We found that when they opted to participate, patients and caregivers generally welcomed the opportunity to speak with us about their experiences, and unburden themselves, however partially, of the weight they shouldered.

### Surrendering to Life and Death: Philosophies of Care on the Ward

Many of the palliative care clinicians we came to know approached the life and death of the body as sacrosanct processes inviolate to human intercession. Echoing Catholic doxa, ideologies of palliative care on the ward entailed reverence for a divine telos and surrender to the “natural” temporalities of life and death, a sentiment captured in the biblical construction uttered by one clinician and for which this article is named: “there is a time to be born and a time to die” (referencing notions from Ecclesiastes 3:2). The seemingly contradictory imperatives of encouraging patients to embrace life *and* accept death that organized care on the ward, we want to suggest, in fact reflect the same moral impulse born from what we have come to think of as the palliative clinicians’ orientation of surrender. “Dignified death”, for these clinicians, entailed *surrendering* to the body’s preordained rhythms; they wanted their patients to accept and embrace the time they had been given on Earth without working to artificially extend or interrupt it.

The work of attuning patients to surrender to the preordained temporalities of life and death was painstaking. In some cases, it entailed bringing patients along to the reality of their situation, helping them to accept their imminent mortality despite a strong desire to cling to life. In other cases, patients pleaded for the doctor to help them die. They were ready to go, even though their body was not. Here, the challenge was to help them live, as comfortably as possible and against their will, alongside the reality of imminent death. When patients achieved a balance—having accepted their mortality but not desiring to hasten death—palliative care providers felt they had done their job well. Palliative clinicians described this process as challenging, but also deeply gratifying. For Dr. Denis, an experienced clinician with a background in anesthesiology, the process entailed “cross[ing] that bridge and connect[ing] with [the patient] such that they achieve peace.” She elaborated, “it is amazing how a patient who has just been given the news that he is no longer eligible for chemotherapy due to a deterioration or progression of the disease—and he comes to me suffering, with uncertainty—how with communication skills and with the most specific words possible, [you can] observe that this suffering transforms into peace, into acceptance.”

The INCan clinicians were committed to ameliorating patient suffering across multiple scales. “For some patients, pain is suffering,” said a clinician named Dr. Mariela. “For others, economic stress [related to the disease and its treatment] causes suffering… So, I think that the approach to the patient is so important and so specialized, it is unique. [The job requires us to] pivot from where we are to where we might be most helpful.” About the subjective and multidimensional nature of suffering, Dr. Molina agreed with her colleague, stating, “[one] normally assumes that the most predominant symptom will be pain … but once you get to know the person—and you get to know them well from the follow-up appointments … There are times when the main symptom or concern is that they have not baptized their child, for example, things that I did not believe were [relevant to medicine] but were causing a lot of emotional distress for the patient.” Clinicians emphasized the importance of tailoring their approach to each patient, invoking the metaphor of cutting and fitting a piece of fabric to make an article of clothing [“*un traje a la medida*”]. Yet even the treatment of a single patient was dynamic, requiring constant modification. Dr. Denis observed, “I [have to adjust] the treatment goals every day because they keep shifting. [One day] the patient might say that she understands [the diagnosis and prognosis] and maybe we even talk about an advance directive, and then [her perspective] shifts into denial, into anger.”

If addressing patient suffering was their guiding objective, the ultimate purpose of this work was to guarantee patients a “dignified death.” For the clinicians, the contours of “dignified death” varied from person to person, according to their wishes, but implicit in this understanding was respect for and surrender to the “natural” rhythms of death, which precluded intervention through medical or other means. This orientation was central to ideologies of healing on the ward, and to the education and socialization of new generations of palliative care clinicians, emerging most forcefully in scenarios that challenged this philosophy, such as assisted dying.

The INCan clinicians were clear that to help a patient die before their time represented a violation of the moral underpinnings of palliative care. When we asked Dr. Tiaca, the Chief of Palliative Care at one public hospital in the capital, about her position on the national debate over the legalization of assisted dying, her retort captured prevailing sentiments in the field. “[Euthanasia] is not my job… we [palliative care professionals] are in favor of life.” If the procedure were to be legalized in the capital, she indicated that she would exercise her right to conscientious objection.

[As doctors], from the moment we take our Hippocratic Oath, we express the care that we are going to give to a patient throughout their entire life trajectory and, obviously, there is something within our code of deontology that states that we are not going to kill people, right? I think we are too green [in Mexico] to be able to adopt euthanasia because we do not even have a universal palliative care system in place. So, most patients request it because they are suffering and if they weren’t suffering, they probably wouldn't be asking for it, right?

Like Dr. Tiaca, other palliative clinicians expressed that once comprehensive palliative care were accessible to all, assisted death would be rendered moot. Reflecting what Kimberly Arkin (2020) observed in French palliative care, several INCan clinicians described patient desires to accelerate death not as genuine expressions of suicidality, but rather as reflections of an abundance of untreated suffering that could be ameliorated through palliative means. Dr. Molina said, “I think that those who promote euthanasia don’t understand palliative medicine and euthanasia is for them an easy way out, when someone is suffering with a terrible insanity of symptoms and they have a pistol nearby, obviously that is the quick and easy way out. It’s easier and faster than recognizing that people have a right to be free of pain and to have access to medications. I believe that those who solicit euthanasia are afraid of how they will die, of being in pain.” When articulating their opposition to assisted dying, clinicians sometimes invoked religious or spiritual reasoning. Dr. Mariela said, “If a patient asks me for euthanasia, I tell them to find another doctor because I don’t kill people for a living. I wouldn’t be able to handle that burden. Independent of the religion I profess, because I am *creyente* [a believer in God], I believe that there is someone above us and that I cannot give or take life. That duty corresponds to the being above us [mortals].”

A minority of INCan clinicians did express support for or openness to assisted dying. Palliative care residents, generally ten to fifteen years younger than the established clinicians, were most sympathetic to this possibility. Moreover, Dr. Andrea, among the most experienced palliative care clinicians at the INCan and a leader in the field, also expressed a less common perspective. “In the case that [medically assisted death] was legal and regulated, I wouldn’t tell a patient no. I’m not in favor of someone living longer than they should. I’m not in favor of human suffering.” Her response departed from prevailing approaches in palliative care, while relying on definitional commitments in the field to ameliorate suffering. But then, she hedged, wondering aloud about the impact of permissive legislation around euthanasia, and abortion, on future generations:

What would we be teaching society? That we [humans] are dispensable? What are [future generations] going to learn if euthanasia is legal? … [Nowadays] babies are made in test tubes. Even if God forbids [me] from having a baby, I can pay for it … I can rent a womb, I can buy an egg, they take it from me and collect sperm … and here you are, you are a son of this family. You arrived not because of God’s will but because of my will. This removes everything magical, mysterious, and natural. And now you're not going to die when it's your turn due to nature's chances, but *you* decide when you’re going to die. That worries me, that lesson for future children. What are those kids going to learn?

Even though she expressed support for the practice, for Dr. Andrea, assisted dying, like abortion, raised fraught questions about the limits of human agency. Her commitment to alleviating suffering conflicted with her belief in the inviolability of the body and its “natural” processes, causing moral doubt and ambivalence about hastened death beyond its legal status.

When patients’ orientation to death resisted surrender, either because they wanted to end their life prematurely or avoid it indefinitely, the project of palliative care was called into question. For this reason, as we elaborate below, Karo’s “death denial” and the possibility of Alejandro’s suicide both occasioned moral distress for the palliative providers, embodying the limits of choice available in Mexican palliative care.

## Karo’s Story: Disavowing Death

We had been observing in Dr. Molina’s consultation room in July of 2023 when Karo, age 44, arrived with her mother. After several rounds of chemotherapy, her oncologist had referred her to palliative care. Karo had opted for a “manejo conjunto”, a treatment of both oncology and palliative care. The tension between Karo and her mother was unmistakable, sucking up all the energy in the cramped room. “The disease is like a prison,” Karo whimpered. “I want to get my independence back.” Dr. Molina told Karo that she could not cure her disease, but that if she decided to accept palliative care, they would work together to control her symptoms and make her more comfortable. Karo and her mother agreed. As they stepped out of the room, Dr. Molina explained to us that it was important to be agile about introducing the topic of death to patients. Karo was not ready to have a conversation about her end-of-life wishes; she was still fighting to regain independence.

When we got a chance to speak in a private corner of the ward, Karo narrated everything that breast cancer had taken from her since her diagnosis five years earlier, one devastating loss after another. First it was her job. She had created a small business, but the chemotherapy, the nausea, and the constant travel from her home state of Jalisco to the INCan in Mexico City had forced her to abandon all she had built. After her job, it was her partner. He had cheated, she said, tears splashing down her face. It was pride that forced her to leave him. She moved back with her mother, doting and generous, but in a suffocating way. So, she had lost her partner, and then her autonomy as well. Before she got sick, we learned, Karo had yearned for a baby, and her illness had robbed her of that dream, too. It wasn’t clear what was left in Karo after all that loss. “I miss my breasts,” she cried, gripping her sunken chest. The cancer had recently migrated to her brain and begun to impair her vision, she said.

If the desire to hasten death threatened the approach of palliative care clinicians, resistance to mortality was also troublesome, as Karo’s case suggests. Karo was new to palliative care, and her struggle with acceptance was to some degree an expected part of the process of coming to terms with terminal illness. At the same time, for the palliative care clinicians her orientation demanded redirection, as she had not yet arrived at a place of peaceful surrender. This was clear enough to us when, after reading the consent form before our interview, Karo recoiled at the study subtitle: “Searching for Dignity at the Ends of Life.” “It’s hard to read that,” she said, her comment demanding restraint in the research encounter and foreclosing certain domains of conversation about fears or plans for death. Instead, when she opted to continue with the interview, Karo took her story back in time, recalling a self before the diagnosis. She insisted that once she had been pretty, pulling up an old photograph on her cell phone. It was hard to see even a trace of the woman in the photograph in the frail person sitting before us, her hair now clipped at the scalp, her frame angular, younger and older all at once.

As the interview progressed, Karo reflected on the losses that had defined the past five years, bringing her story forward with frequent references to the time before illness struck. “My life before this illness was completely different,” she said, her voice breaking. “[I was] totally independent physically and financially. In fact, for many years, I lived alone with my partner, everything was great … everything changed, and little by little, since then, I have seen that my life has become more and more dependent … that all that independence has been lost little by little.” While she hoped for a miracle, her immediate goals were to find relief from the agonizing pain and exhaustion wrought by the cancer and chemotherapy, respectively. Most importantly, though, Karo wanted to break free from her mother and become independent again.

When we first met Karo during her intake session with Dr. Molina, we had no way of knowing how little time she had left. She looked frail, but she spoke with conviction and clarity. With time, we learned to read the doctors’ words and body language for clues about patients’ prognoses. Dr. Molina generally waited to introduce the topic of death until her patients indicated a degree of awareness about their mortality, or until they received bad news in the Oncology Department. But even then, these were not easy conversations. “I touched on [a hard topic] with the patient today,” Dr. Molina said about this process, “and you might have noticed my voice trembling a bit, because it’s hard to talk about these things, so the idea is to enter the fog little by little. When we have the conversation and get to talking, I try to bring up everything that needs to be discussed because then it’s case-closed, because if they are alive, we focus on quality of life. We have to talk about death, but the idea is not to keep jabbing the patient with it.”

After the interview, we saw Karo just one more time on a follow-up visit to the INCan in which she sought relief from a particularly uncomfortable symptom. We learned later that she died at home in Jalisco on October 19, 2023, exactly three months after our conversation on the palliative care ward. By her mother’s account, it had been a harrowing process. That week, she had taken her daughter for what would be her final round of chemotherapy. “It was the worst chemo,” she said. “It caused so much damage, and she had broken her leg [because her bones were brittle], so she was dealing with that pain, it was horrendous to [watch her] in so much pain.” Karo’s mother rushed to the palliative care ward for answers. When a clinician called her back, she was desperate.

I'm just coming down from oncology… My daughter is in the terminal phase. My daughter is in agony. My daughter is already in so much pain. What I want is for you to tell me, what am I going to do? How am I going to adjust the medication? [I want you] to tell me how I’m going to help her die! Tell me! What should I do?

The doctor prescribed Fentanyl, explaining how Karo’s mother was to administer the medication intravenously every six to eight hours. “I felt like I couldn’t breathe,” she said, “I was so desperate.” Karo’s mother explained that Karo had wanted to live. “[She told me] I don't want to die, I don’t want to die, I don’t want to die! She was afraid of dying… She fought, fought, fought, and she took everything they gave her. Do you know how many times they pricked her [with a needle] last time, on the ward? 48 times!”

As death drew closer, Karo continued to resist. Her mother wept over the phone, recounting the final moments:

[Karo] said, ‘Mami, I don’t want to die. I’m scared, Mami.’ And I said, ‘Look, honey. Don’t be afraid. Death is beautiful. We should love death because it takes the most cultured people like you, the most beautiful, the ugliest, the children, the elderly. Death is beautiful, honey because it doesn’t discriminate, don’t be afraid, look, your grandparents, your aunts, and your daddy are there. They’re going to throw you a big party when you arrive. Remember us, honey, when you’re in God’s paradise. And don’t be afraid, honey.

Karo had asked to be cremated. Her mother said that today she keeps her ashes nearby. “She’s my only company now.”

Karo resisted death through her final moments, disavowing the orientation her palliative care clinicians worked to impart. Her refusal, or inability, to surrender to death departed sharply from the palliative model of care, precluding, in their definition, a “dignified” passing. At the same time, she eventually learned to accept her mother’s assistance, leaving this world in her loving embrace.

## Alejandro’s Story: Suicidal Specters

When Alejandro, a thirty-year-old patient on the INCan palliative care ward passed away at home in March of 2024, his medical team feared that he had died by suicide, defined on the ward as an unequivocally “bad death.” Their concern was understandable given that Alejandro had struggled with suicidal ideation since his cancer diagnosis: osteosarcoma with metastases in the lungs, spine, and hips. Early on he had shared a particularly harrowing incident with his palliative team that left them hypervigilant. He had been standing on a bridge above traffic and planned to jump, leaving this world and his troubled place in it behind, but his mother didn’t know where he was, and that thought held him back. With the support of palliative care, Alejandro’s will to live had increased, at least for stretches. But at times, the physical pain of his cancer was too agonizing to bear. On his final trip to the INCan in January of 2024, two months before he died, he had reportedly begged his mother to kill him with a knife.

After learning of Alejandro’s death, the palliative care team called his mother several times until they finally reached a different family member. His mother hadn’t been able to get out of bed for a month, her grief incapacitating, but the family member assured them that Alejandro had died naturally. The team’s questions were mostly answered, although his psychologist, Dr. Ortega, still harbored some doubts. At the same time, our own questions kept multiplying. Considering Alejandro’s looming mortality and the anguish that defined his final months, why had the palliative care clinicians been so anxious that he had accelerated his death? Given his terminal diagnosis, if he *had* accelerated his death, wouldn’t this be different, conceptually and ethically, from suicide?^14^

Everyone on the ward had known that for Alejandro, it was only a matter of time. The cancer had been growing in his body for a few years by the time he was finally referred to the INCan, five hours from his home state of Guerrero, in the fall of 2022, first for oncology treatments, and later for palliative care. What was originally an osteosarcoma in his left forearm had metastasized, slowly at first, and then quickly. By winter of 2024 he was paralyzed from the waist down by a tumor outgrowth that cut through his spine. Beyond the cancerous lesions that had overtaken his body, Alejandro’s was a complicated case, one that demanded extra attention and commitment on behalf of the palliative care team. Alejandro had grown up gay in a conservative state and had been subjected to bullying since childhood. He came from a poor family, one of several siblings. His father had been his refuge, the person who quietly shielded him from the homophobia he encountered even within his own family. When his father died suddenly in a traffic accident in 2017, it was a hard blow for everyone, but especially for Alejandro. It was only then that he found the courage to come out to his mother, whose response only amplified his sense of loss: “No wonder God took your dad away. He wanted to spare him this unpleasant reality.” Despite her scornful comment, it was impossible to miss her commitment to Alejandro. Every time we saw her on the palliative care ward, she was distraught, her doting care a testament to her abiding love for her son. What she didn’t know was that Alejandro had also been living with HIV; this part he had been too ashamed to tell her.

Over the course of almost a year, Dr. Molina, Alejandro’s anesthesiologist, Dr. Ortega, his psychologist, and the rest of his palliative care team monitored him closely, working carefully to treat his depression and excruciating pain during monthly check-ups, and as their relationship deepened and death drew near, encouraging him to unburden himself of the painful secret he kept from his mother so he could pass peacefully. When he finally mustered the courage to tell her, she had been gracious and understanding. Eventually, the team instructed Alejandro not to return to the ward in person because the risk of  dying en route was too high. Alejandro’s mother would travel on his behalf to collect his medications, and the team would reach him by phone, until the end.

In the final months of his life, Alejandro seemed to be oriented to death in ways that his palliative care team deemed ideal. He had accepted that he was going to die, and he had made specific requests. For his wake, he wanted to be dressed in the T-shirt that bore his late father’s face, the same one that his mother sometimes wore to the hospital. When we interviewed him, Alejandro wasn’t afraid to talk about dying the way Karo had been. He told us that he wanted to die at home, surrounded by his mother, his siblings, and his partner. Not only had he come to terms with his mortality, but toward the end of his life Alejandro’s suicidality had also seemed to wane. He had even sworn to Dr. Molina that he would not take his own life because of the painstaking care the palliative team, and particularly his psychologist Dr. Ortega, had invested in him.

In January of 2024, during Alejandro’s last visit to the INCan, the team had managed to control his excruciating pain. He lay in bed, paralyzed, his voice feeble and his skin pale, beads of sweat gathering on his forehead. When the team discharged him from the hospital, they knew that the pain control they had achieved wouldn’t last long, and they referred him to a local hospital in Guerrero to save his mother the long trip to the INCan. But she and Alejandro felt abandoned by that suggestion, so the team kept caring for him from afar. Dr. Ortega had been in Europe for a conference when Alejandro wrote to him for the last time. He missed the message. Back in Mexico, he tried calling Alejandro, but there was no answer. After speaking with Dr. Molina, he learned that Alejandro’s mother never arrived at the hospital the week before to collect her son’s pain medications, and they both grew suspicious. “We started calling, calling, calling, calling, calling,” Dr. Ortega said. “Well, I think it took a long time, more than a month or two months, I don’t remember, until … a relative [answered the phone]. She said he had passed away.”

Alejandro’s doctors wanted to believe that he had died naturally, but it didn’t add up. Dr. Ortega had serious doubts:

I was questioning and I felt guilty because I was attached to him, and I had gone on this trip, and I told myself I’d be back in two or three days … and I would contact him. But I grew suspicious, I started wondering what had happened … [Alejandro] was constantly thinking about suicide. [It occurred to me that] it would have been difficult for him to commit suicide because of the position he was in. I mean, he couldn’t walk … he had no movement in his legs, and he was very heavy. Somebody would have had to help him.

Amidst the questioning, remorse started to creep in, prompting Dr. Ortega to reflect on the limits of his role as a palliative care clinician.

It was the first time while working in palliative care that I questioned whether one of my patients had committed suicide. So, I started to [think about] how far I [can] go [as a doctor], I mean how far I go in my involvement, in my commitment to the patient …

There is no way to know for sure what happened to Alejandro, if he drifted away in his sleep, or if he found a way to end it. Alejandro’s mother never responded to our request for a follow-up interview, and Dr. Molina thought it best for us to let her grieve. For the palliative care clinicians, suicide was an unthinkable option, one that existed outside of and against palliative care, independent of the circumstances. Their work was predicated on an assumption they took to be self-evident: “that life is worth living, that life itself is its own value” (Stevenson, 2014: 8). The haunting possibility that Alejandro had taken his own life, or that someone had helped him die, indicated, for them, that his relationship to mortality had drifted from the orientation they had worked so hard to achieve, that he had lost the will to live. The specter of his suicide was hard to bear. It violated a moral imperative against hastened death that is definitional to Mexican palliative care, but more importantly, it suggested that Alejandro had been suffering uncontrollably and that they had failed to care for him.

## Discussion and Conclusion: Paradoxes of Choice

For the INCan palliative care clinicians, both cases we have sketched here represent opposite expressions of the same resistance to surrender to one’s “time to die.” The problem was not that the patients died, but rather *how* they died: one fighting against the inevitable, and the other desiring to take control of a process that does not, according to the clinicians, correspond to human agency. Karo’s struggle to accept her mortality demanded intervention and attunement such that she could achieve the kind of death her clinicians considered desirable. Denial of death, whether it took the form of cognitive refusal, as in Karo’s case, or a preference for indefinite medical intervention, as in other cases outside the scope of this article, constituted a logical impossibility within the palliative orientation of surrender. At the other end of the spectrum, Alejandro's potential suicide also threatened this orientation, provoking questions about the limits of human interference in the dying process, and the inability of palliative clinicians to know what really happens in a patient’s final moments. The idea that Alejandro had taken his own life, much like patient desires to hasten death through euthanasia, represented for the clinicians an assault on the fundamental principles of palliative care. Even though the clinicians considered Karo and Alejandro’s deaths to be problematic, they remained compassionate and caring for each of these patients, never chastising them or making them feel guilty. Their clinical experience made it clear that not all patients could access the kind of “dignified death” that Araceli had, even as they strove for this outcome in all their work.

As a result of the Advance Directive Law, terminally ill patients in the Mexican capital today have an expanded set of  end-of-life choices. In public palliative care wards like the INCan, clinicians communicate with their patients in clear and honest language about their disease and prognosis; they afford a space and a vocabulary through which to speak openly about death; and they offer patients a concrete set of choices about whether and when to stop curative measures, what kinds of pain management and psychological interventions they desire, as well as where they want to die and with whom. And yet, even as palliative care in this context creates the conditions for certain choices, it also positions itself against the implications of choice itself, making certain choices unthinkable (e.g., euthanasia, suicide, or death denial). The public sphere of Mexican palliative care, in short, restores to patients a sense of agency and choice at the end of life—particularly in the face of paternalistic medical systems and the threat of therapeutic obstinacy—but it also, ultimately, posits a different reading of what human agency is or should be.

## Data Availability

No datasets were generated or analyzed during the current study.

## Footnotes

1 To protect participant privacy, we use pseudonyms for all people who appear here. Moreover, we have altered certain identifying details, such as state of origin or role within the hospital, in cases where this information might reveal someone’s identity.

2 The first author designed this study around the concept of “dignified death” in light of how local activists define their work. Nevertheless, the study does not rely on a predetermined definition of what “dignified death” entails. Rather, it was designed in part to understand how local actors—activists, clinicians, patients, and ordinary people—imagine and strive to accomplish this outcome.

3 The first author is the PI of the study from which data for this article are drawn, and the second author is a Research Associate on the project.

4 In Mexico, “dignified death” activists use a range of terms to advocate for self-determination over how one dies, including “muerte medicamente asistida” (medically assisted death) and “suicidio medicamente asistido” (medically assisted suicide). In this article we use the English terms “assisted dying” and “assisted death” to describe the process whereby a physician prescribes a lethal medication to a terminally ill patient who wishes to end their suffering. For ease of language, we occasionally also use the term “euthanasia.”

5 In 2023, for instance, the Mexican Bishop of Culiacán publicly rejected a legal initiative to change the Mexican General Health Law to permit medically assisted dying, equating the practice with murder in an open letter: “from the standpoint of faith, therefore, we reaffirm that it is not lawful to seek euthanasia, which eliminates the life of a person, nor is it lawful to carry out legislative or judicial acts that attempt to legitimize an act that is intrinsically contrary to respect for human life as a gift from God” (Herrera Quiñónez 2023).

6 Despite the political and social power of Catholic prohibitions on euthanasia in Mexico, popular opinion reflects support for its legalization. A national survey conducted by Ordóñez-Vázquez and Ortiz-Millán, revealed that 50.2% of respondents were in favor of the right to euthanasia among the terminally ill. Moreover, Derecho a Morir con Dignidad México (DMD), a civil association in Mexico City dedicated to promoting dignified death, conducted a separate national survey on opinions on euthanasia in 2022 in which an overwhelming 72.7% of respondents stated that euthanasia should be legalized.

7 We are inspired here by Lisa Stevenson’s (2014) pathbreaking work on Inuit suicide in the Canadian Arctic, and specifically her critical reading of the “suicide apparatus” that approaches life as an ultimate good, independent of the circumstances and desires of those leading it.

8 However, the Euro-American terror of dependency and physical decline is not universal, and in some contexts, elderly people welcome new social roles defined by increased care and assistance from kin (Lamb, 2014, 2018).

9 In this article, we cite just one interview with a palliative care doctor from a Mexico City public hospital other than the INCan.

10 Palliative care services are free to oncology patients at the INCan; however, if a patient is no longer receiving oncological treatment, they must pay for palliative care.

11 We obtained IRB approval from the University of Oklahoma and from Southern Methodist University, as well as from the INCan hospital ethics committee.

12 All palliative care patients older than 18, who had a terminal diagnosis and were aware of that diagnosis, and who were determined by the hospital palliative care team to be in an adequate psychological and physical state to consent to and participate in research were eligible. Patients suffering from advanced dementia, Alzheimer’s, or other conditions that impaired cognition to the degree that they could not provide informed consent were excluded from the study.

13 Imada restrained entirely from conducting oral histories among survivors of medical incarceration in the Hawaiian Leper colony today known as Kalaupapa National Historical Park. Total restraint, in that context, was as an ethical way to break from a research enterprise that risked reproducing legacies of the very colonial health projects under study.

14 We are inspired here by Anita Hannig’s (2019) article on the moral and conceptual difference between suicide and medical aid-in-dying.

## References

[CR1] Álvarez Del Río, A. (2009). Algunos avances en la regulación sobre la eutanasia en América Latina: el caso de Colombia y México. *Perspectivas Bioéticas,**14*(26–27), 162–167.

[CR2] Andrade, F. (2009). Estimating diabetes and diabetes-free life expectancy in Mexico and sevan major cities in Latin America and the Caribbean. *Revista Panamericana De Salud Pública,**26*(1), 9–16. 19814876 10.1590/s1020-49892009000700002 PMC4059400

[CR3] Angel, J. L., Vega, W., & López-Ortega, M. (2017). Aging in Mexico: Population trends and emerging issues. *The Gerontologist,**57*(1), 153–162. 27927730 10.1093/geront/gnw136 PMC5881744

[CR4] Arkin, K. A. (2020). What can words do? Debating a “Good” death in French. *Anthropology Quarterly,**93*(2), 177–204.

[CR5] Carlos, H. B., Werutsky, G., Mohar, A., Ferrigno, A. S., Müller, B., Bychkovsky, B. L., Castro, C. J., Uribe, C. J., Villarreal-Garza, C., Soto-Perez-de-Celis, E., Gutiérrez-Delgado, F., Kim, J. S., Ismael, J., Delgado, L., Santini, L. A., Teich, N., Chavez, P. C., Liedke, P. E., Exman, P., … Cazap, E. (2021). Cancer control in Latin America and the Caribbean: Recent advances and opportunities to move forward. *The Lancet Oncology,**22*, e474–e487. 34735817 10.1016/S1470-2045(21)00492-7

[CR6] Biehl, J. (2005). *Vita: Life in a Zone of Social Abandonment*. University of California Press.

[CR7] Buch, E. (2018). *Inequalities of aging: Paradoxes of independence in American Home Care*. New York University Press.

[CR8] Buch, E. (2013). Senses of care: Embodying inequality and sustaining personhood in the home care of older adults in Chicago. *American Ethnologist,**40*(4), 637–650. 26401062 10.1111/amet.12044 PMC4577066

[CR9] Buchbinder, M. (2018). Choreographing death: A social phenomenology of medical aid-in-dying in the United States. *Medical Anthropology Quarterly,**32*(4), 481–497. 30014621 10.1111/maq.12468

[CR10] Buchbinder, M. (2020). *Scripting death: Stories of assisted dying in America*. University of California Press.

[CR11] Catholic Church. Catechism of the Catholic Church. 2nd ed., Our Sunday Visitor, 2000.

[CR12] Covarrubias-Gómez, A., Otero-Lamas, M., Templos-Esteban, L. A., & Soto-Pérez-de-Celis, E. (2019). Antecedentes de la medicina paliativa en México: educación continua en cuidados paliativos. *Revista Mexicana de Anestesiología,**42*(2), 122–128.

[CR13] Das, V., & Han, C. (2016). *Living and dying in the contemporary world: A compendium*. University of California Press.

[CR14] DelVecchio-Good, M. J., Gadmer, N. M., Ruopp, P., Lakoma, M., Sullivan, A. M., et al. (2004). Narrative Nuances on Good and bad deaths: Internists’ tales from high technology work places. *Social Sciences & Medicine,**58*(5), 939–953. 10.1016/j.socscimed.2003.10.043 14732607

[CR15] Espinosa, A. R. (2016). Por el Derecho a Morir con Dignidad, A.C. Encuesta Nacional Sobre Muerte Digna

[CR16] Fajardo-Chica, D. (2020). Sobre el concepto del dolor total. *Salud Pública,**22*(3), 368–372. 10.15446/rsap.V22n3.84833 36753165 10.15446/rsap.V22n3.84833

[CR17] Gerson, S. M., Koksvik, G. H., Materstvedt, L. J., & Clark, D. (2020). The relationship of palliative care with assisted dying where assisted dying is lawful: A systemic scoping review of the literature. *Journal of Pain and Symptom Management,**59*(6), 1287–1303. 31881289 10.1016/j.jpainsymman.2019.12.361 PMC8311295

[CR18] Gómez Flores L. (2023). En 15 años, más de 20 mil personas han firmado su voluntad anticipada. La Jornada https://www.jornada.com.mx/notas/2023/07/12/capital/en-15-anos-mas-de-20-mil-personas-han-firmado-su-voluntad-anticipada/

[CR19] González, C., Méndez, J., Romero, J. I., Bustamante, J., Castro, R., & Jiménez, M. (2012). Cuidados paliativos en México. *Revista Médica Del Hospital General,**75*(3), 173–179.

[CR20] Hannig, A. (2019). Author(iz)ing death: Medical aid-in-dying and the morality of suicide. *Cultural Anthropology,**34*(1), 53–77.

[CR21] Hannig, A. (2022). *The day I die: The untold story of assisted dying in America*. Sourcebooks.

[CR22] Herrera Quiñónez, J. J. (2023). Comunicado acerca de la reforma legislative sobre la eutanasia. Culiacán, Sinaloa, November 27. https://cem.org.mx/comunicado-acerca-de-la-reforma-legislativa-sobre-la-eutanasia/

[CR23] Huertas, L. A., Rodríguez-Mayoral, O., García-Salamanca, M. F., Monreal-Carrillo, E., & Allende-Pérez, S. (2022). Suicide in cancer patients undergoing palliative care: A report of two cases. *Salud Mental,**45*(2), 89–93.

[CR24] Imada, A. L. (2022). *An Archive of Skin*. Disability and Life-Making During Medical Incarceration. University of California Press.

[CR25] Investigación en Salud y Demografía, S.C. (Insad). (2022). Encuesta nacional "Por el Derecho a Morir con Dignidad, México 2016, 2022.” https://dmd.org.mx/wp-content/uploads/2023/07/Principales-resultados-Comparacion-Primera-y-Segunda-encuesta-DMD-2016-2022.pdf

[CR26] Instituto Nacional de Estadística y Geografía. (INEGI). (2025). Estadísticas de defunciones registradas (EDR). https://www.inegi.org.mx/contenidos/saladeprensa/boletines/2025/edr/edr2024_en-jun.pdf

[CR27] Kaufman, S., & Morgan, L. (2005). The anthropology of the beginnings and ends of life. *Annual Review of Anthropology,**34*, 317–341.

[CR28] Kaufman, S. (2005). …*And a Time to Die: How American hospitals Shape the End of Life*. Scribner.

[CR29] Kaufman, S., & Fjord, L. (2011). Medicare, ethics, and reflexive longevity: Governing time and treatment in an aging society. *Medical Anthropology Quarterly,**25*(2), 209–231. 21834359 10.1111/j.1548-1387.2011.01150.x PMC3555685

[CR30] Knaul, F. M., Farmer, P. E., Krakauer, E. L., De Lima, L., Bhadelia, A., Kwete, X. J., Arreola-Ornelas, H., Gómez-Dantés, O., Rodriguez, N. M., Alleyne, G. A., & Connor, S. R. (2018). Alleviating the access abyss in palliative care and pain relief—An imperative of universal health coverage: the Lancet Commission report. *The Lancet,**391*(10128), 1391–1454. 10.1016/S0140-6736(17)32513-8 29032993

[CR31] Koksvik, G. H. (2020). Medically timed death as an enactment of good death: An ethnographic study of three European intensive care units. *Omega*. 10.1177/0030222818756555 10.1177/0030222818756555 29402160

[CR32] Krawczyk, M. (2017). Approaching Death: Informed Consent and Research Relationships at the End of Life. *Medicine Anthropology Theory*. Dissertating 4(1).

[CR33] Lamb, S. (2014). Permanent personhood or meaningful decline? Toward a critical anthropology of successful aging. *Journal of Aging Studies,**29*, 41–52. 24655672 10.1016/j.jaging.2013.12.006

[CR34] Lamb, S. (2018). On being (not) old: Agency, self-care, and life-course aspirations in the United States. *Medical Anthropology Quarterly,**33*(2), 263–281. 10.1111/maq.12498 30575125

[CR35] Lawton, J. (2001). Gaining and maintaining consent: Ethical concerns raised in a study of dying patients. *Qualitative Health Research,**11*(5), 693–705. 11554196 10.1177/104973201129119389

[CR36] LemosDekker, N. (2019). Standing at the Doorstep: Affective Encounters in Research on Death and Dying. In: Stodulka, T., Dinkelaker, S., Thajib, F. (eds) Affective Dimensions of Fieldwork and Ethnography. Theory and History in the Human and Social Sciences. Springer, Cham. 10.1007/978-3-030-20831-8_18

[CR37] Mansilla-Olivares, A., López-Bárcena, J., Valenzuela-Gómez-Gallardo, F., Rojo-Medina, J., Meneses-González, F., & Vázquez-De Anda, G. F. (2019). National Academy of Medicine of Mexico posture with regard to end-of-life decisions. *Gac Mexico.,**154*(6), 628–31.

[CR38] McNamara, B. (2004). Good enough death: Autonomy and choice in Australian palliative care. *Social Science & Medicine,**58*(5), 929–938. 14732606 10.1016/j.socscimed.2003.10.042

[CR39] de Oca, M., & Lomelí, G. (2006). Historia de los Cuidados Paliativos. *Revista Digital Universitaria,**7*(4), 2–9.

[CR40] Morgan, L. (2009). *Icons if life: A cultural history of human embryos*. University of California Press.

[CR41] Nishimura, M., Dening, K. H., Sampson, E. L., de Oliveira Vidal, E. I., de Abreu, W. C., Kaasalainen, S., Eisenmann, Y., Dempsey, L., Moore, K. J., Davies, N., & Bolt, S. R. (2022). Cross-cultural conceptualization of a good end of life with dementia: a qualitative study. *BMC Palliative Care,**21*(1), 106–118. 35676673 10.1186/s12904-022-00982-9 PMC9175529

[CR42] Norwood, F. (2020). *The maintenance of life: Preventing social death through Euthanasia talk and end-of-life care—Lessons from the Netherlands*. California Academic Press.

[CR43] Norwood, F. (2018). The new normal: Mediated death and assisted dying in the United States. In A. C. G. Robben (Ed.), *A companion to the anthropology of death. *Wiley.

[CR44] Ordóñez-Vázquez, N. A., & Ortiz-Millán, G. (2023). A regulamentação da morte com dignidade no México: uma questão pendente. *Revista Colombiana de Bioética,**18*(2), e3870.

[CR45] Ricahrds, N., & Krawcyzk, M. (2021). What is the cultural value of dying in an era of assisted dying? *Medical Humanities,**47*(1), 61–67. 10.1136/medhum-2018-011621 31350304 10.1136/medhum-2018-011621

[CR46] Robledo, M. G., Lopez Ortega, M., & Arango Lopera, V. E. (2012). The state of care in Mexico. *Current Translational Geriatrics and Experimental Gerontology Reports,**1*(4), 183–189.

[CR47] Roberts, E. (2012). *God’s laboratory: Assisted reproduction in the Andes*. University of California Press.

[CR48] Rodríguez-Mayoral, O., Ascencio-Huertas, L., Verástegui, E., Delgado’Guay, M. O., & Allende Pérez, S. (2019). The desire to hasten death in advanced cancer patients at a Mexican palliative care service. *Salud Mental,**42*(3), 103–109.

[CR49] Rouse, C. (2008). “If she’s a vegetable, we’ll be her garden”: Embodiment, transcendence, and the citations of competing cultural metaphors in the case of a dying child. *American Ethnologist,**31*(4), 514–529.

[CR50] Samuels, A. (2023). Silence at the end of life: Multivocality at the edges of narrative possibility. *American Anthropologist,**125*(4), 892–895. 10.1111/aman.13922

[CR51] Samuels, A., & Dekker, N. L. (2023). Palliative care practices and policies in diverse socio-cultural contexts: Aims and framework of the ERC globalizing palliative care comparative ethnographic study. *Palliative Care and Social Practice*. 10.1177/26323524231198546 10.1177/26323524231198546 PMC10496469 37706167

[CR52] Seale, C., & van der Geest, S. (2004). Good and bad death: Introduction. *Social Science & Medicine,**58*(5), 883–885. 10.1016/j.socscimed.2003.10.034 14732602 10.1016/j.socscimed.2003.10.034

[CR53] Singer, M., Baer, H., Long, D., Pavlotski, A., & Landham. (2025). *Fourth edition, introducing health anthropology: A discipline in action*. Rowman & Littlefield.

[CR54] Singer, M. (2020). Deadly companions: COVID-19 and diabetes in Mexico. *Medical Anthropology: Cross-Cultural Studies in Health and Illness,**39*(8), 660–665. 10.1080/01459740.2020.1805742 33064573

[CR55] Stevens, G., Dias, R. H., Thomas, K. J. A., Rivera, J. A., Carvalho, N., et al. (2008). Characterizing the epidemiological transition in Mexico: National and subnational burden of disease, injuries, and risk factors. *PLoS Medicine,**5*(7), Article e163. 10.1371/journal.pmed.0050125 10.1371/journal.pmed.0050125 PMC2429945 18563960

[CR56] Stevenson, L. (2014). *Life beside itself: Imagining care in the Canadian Arctic*. University of California Press.

[CR57] Stonington, S. (2015). On the (f)utility of pain. *The Lancet, 385*(9976), 1388–1389 10.1016/S0140-6736(15)60708-5 25890410

[CR58] Timmermans, S. (1999). *Sudden death and the myth of CPR*. Temple University Press.

[CR59] Van der Geest, S. (2004). Dying peacefully: Considering good death and bad death in Kwahu-Tafo. *Ghana. Social Science and Medicine,**58*(5), 899–911. 14732604 10.1016/j.socscimed.2003.10.041

[CR60] Vasquez, E. E. (2021). Detecting diabetes risk: Philanthrocapitalism, diagnostic innovation and epistemic power in Mexico. *Medicine Anthropology Theory,**8*(2), 1–22.

[CR61] van der Geest, S., & Finkler, K. (2004). Hospital ethnography: Introduction. *Social Science & Medicine,**59*(10), 1995–2001. 15351467 10.1016/j.socscimed.2004.03.004

[CR62] Long, D., Hunter, C., & van der Geest, S. (2008). When the field is a ward or a clinic: Hospital ethnography. *Anthropology and Medicine,**15*(2), 71–78. 27269224 10.1080/13648470802121844

[CR63] Wood, J. (2022). Cicely Saunders, “total pain” and emotional evidence at the end of life. *Medical Humanities,**48*(4), 411–420. 10.1136/medhum-2020-012107 33980664 10.1136/medhum-2020-012107

